# Very rare combination of Pierre Robin sequence with patent ductus arteriosus, severe persistent pulmonary hypertension, and sepsis in an Afghan neonate: a case report and literature review

**DOI:** 10.1093/omcr/omaf235

**Published:** 2025-11-26

**Authors:** Mansoor Aslamzai, Turyalai Hakimi, Abdul Hakim Mukhlis, Esrar Ahmad Mansoori

**Affiliations:** Department of Neonatology, Kabul University of Medical Sciences “Abu Ali Ibni Sina” (KUMS), 3rd district, Kabul 1003, Afghanistan; Department of Pediatrics Surgery, KUMS, 3rd district, Kabul 1003, Afghanistan; Teaching Assistant, Department of Neonatology, KUMS, 3^rd^ district, Kabul 1003, Afghanistan; Student at the Faculty of Medicine, KUMS, 3rd district, Kabul 1003, Afghanistan

**Keywords:** Pierre Robin sequence, congenital heart diseases and infection

## Abstract

Pierre Robin sequence (PRS) is a rare congenital abnormality that may complicate serious circumstances in infants. We report a very rare case of PRS in a five-day-old Afghan girl, accompanied by patent ductus arteriosus (PDA), severe persistent pulmonary hypertension of the newborn (PPHN), perinatal asphyxia, sepsis, and low birth weight. The integration of these comorbidities with PRS makes the case noteworthy. The infant was initially admitted due to perinatal asphyxia and hypothermia and, was discharged in stable condition the next day. On the fifth day of life, she was readmitted with a diagnosis of PDA, severe PPHN, and sepsis, and received treatment for these conditions. Finally, the newborn died from cardiopulmonary arrest resulting from respiratory failure caused by severe airway obstruction and comorbidities. PRS may predispose the neonate to PDA, PPHN, perinatal asphyxia and infection. Consequently, it is important to treat coexisting morbidities that worsen prognosis, especially in resource-limited settings.

## Background

Pierre Robin sequence (PRS) is a combination of congenital anomalies, including micrognathia, glossoptosis, and airway obstruction [1, 2]. There is a range in the incidence of PRS from 1/5000 to 1/85000 live births with equal prevalence in both sexes [3, 4]. Although the exact cause of PRS is unknown, genetic factors may play a role. PRS is characterized and diagnosed by the clinical triad of micrognathia, or small jaw; glossoptosis, or abnormal posterior displacement of the tongue; and airway obstruction [1, 5]. Micrognathia is evident at birth and is the diagnostic hallmark [5]. The majority of PRS patients are managed conservatively, with airway application, lateral and prone positioning, and intubation. Approximately 10% of PRS patients require surgery. Mandibular distraction osteogenesis in order to extend the mandible and improve the posterior tongue base position, tracheostomy, and tongue-lip adhesion are surgical alternatives [6–8]. There is limited data in the current literature regarding the simultaneous occurrence of PRS with patent ductus arteriosus (PDA), severe persistent pulmonary hypertension of the newborn (PPHN), perinatal asphyxia, sepsis, and low birth weight; therefore, this complex case is reported to enhance our understanding of such very rare associations.

## Case report

**Figure 1:**
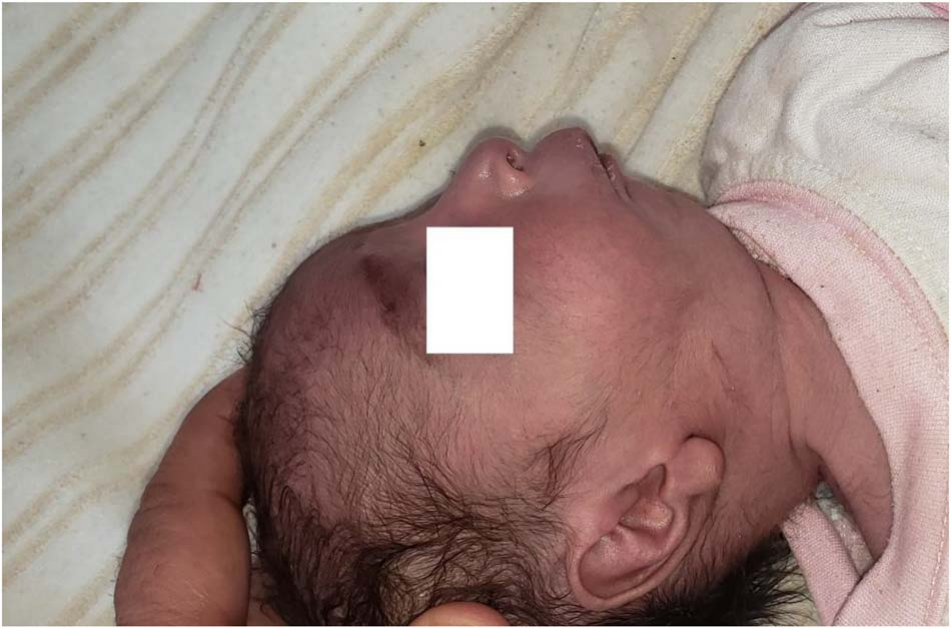
The image shows micrognathia, or underdeveloped mandible, in the index neonate which is the hallmark of Pierre Robin sequence.

A five-day-old girl was admitted to the Neonatal Unit of Maiwand Teaching Hospital due to respiratory distress, sucking failure, and bluish discoloration of skin and mucous membrane which, had appeared a few hours prior to admission. She was born by cesarean delivery at 37 weeks of gestation with a birth weight of 2.3 kg and a length of 52 cm to a 35-year-old multigravida mother at a tertiary hospital. The baby had no respiration or cry at birth with an Apgar score of 4/10 and 6/10 at the 1st and 5th minutes of life, respectively. Resuscitation was performed at the place of delivery, and she was discharged home in good condition after 24 h of admission. The mother was healthy throughout her pregnancy and did not receive any teratogenic medications or radiation. The baby had no history of trauma related to the birth. One of the infant’s sisters has Pierre Robin sequence but is otherwise healthy. Parental consanguinity was confirmed by the parents’ status as third-degree relatives. On the fifth day of life, general physical examination revealed lethargy, rectal temperature of 34°C, respiratory rate of 77/min, heart rate of 170/min, blood pressure of 70/40 mm Hg, weak primitive reflexes, central cyanosis, and oxygen saturation of 73%. Additionally, the infant was found to have micrognathia (a small lower jaw) (Fig. 1) and

**Figure 2:**
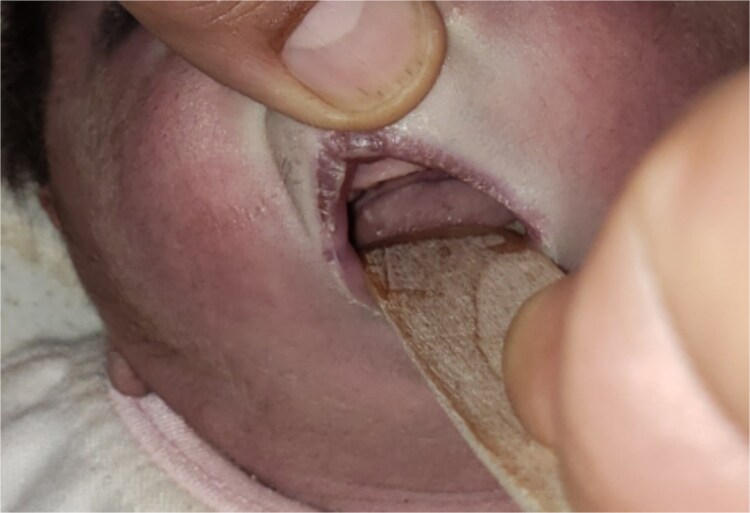
This image demonstrates glossoptosis in the current case which is another key finding of Pierre Robin sequence. The base of the tongue is displaced toward the pharynx due to underdeveloped mandible.

glossoptosis (backward displacement of the tongue) (Fig. 2). These anomalies contributed to airway obstruction, fast breathing, and subcostal and intercostal retractions. There was no evidence of meconium staining on the infant’s mucous membranes or skin. The blood analysis demonstrated a hemoglobin of 13.1 g/dl, a red blood cell count of 3.8 million/mm^3^ (mean corpuscular volume of 95 fl, mean corpuscular hemoglobin of 35 pg), a white blood cell count of 20 500/mm^3^ (46% polymorphs, 49% lymphocytes, 2% eosinophils, 1% monocytes, and 2% basophils), a platelet count of 161 000/mm^3^, C-reactive protein (CRP) of 12 mg/dl, and blood glucose of 89 mg/dl. PRS, neonatal sepsis and low birth weight were diagnosed based on abnormal clinical and laboratory findings, such as micrognathia, glossoptosis, airway obstruction, lethargy, sluggish primitive reflexes, hypothermia, fast breathing, and leukocytosis, an elevated level of CRP and 2.3 kg of birth weight. The patient was placed in prone and lateral positions, provided with an oropharyngeal airway, oxygen therapy at 2 L/min, intravenous maintenance fluids and electrolytes, rewarming, and intravenous antibiotics including Cefotaxime and Ampicillin. The combination of antibiotic therapy was based on the guideline of neonatal unit to treat gram negative and positive bacteria. Following the above management, hypothermia and bluish discoloration resolved, respiratory distress was decreased and oxygen saturation reached to 88%. Doppler echocardiography on the second day of admission showed a 3.4 mm patent ductus arteriosus (PDA) (Fig. 3), severe persistent pulmonary hypertension of the newborn (PPHN) with a peak pressure gradient of 67 mmHg (Fig. 4), dilated right atrium and ventricle (Fig. 5) and severe tricuspid regurgitation (Fig. 6). Ultimately, the diagnosis of PRS accompanied by PDA, severe PPHN, perinatal asphyxia, sepsis, and low birth weight (LBW) was confirmed. Therefore, diuretic therapy and restriction of maintenance fluid were recommended as medical care for PDA. Along with managing neonatal sepsis, minimal handling, sildenafil for pulmonary vasodilatation, and respiratory support were used to treat severe PPHN. Inhaled nitric oxide (iNO), the most effective pulmonary vasodilator, and extracorporeal membrane oxygenation (ECMO) are not available in our country. At this stage, respiratory distress was increased, and oxygen saturation was dropped to 65% due to glossoptosis, critical respiratory obstruction, PDA, severe PPHN and sepsis. Arterial blood gas analysis showed a PaCO₂ of 70 mmHg, a PaO₂ of 40 mmHg, and a pH of 7.20 on 100% oxygen, indicating respiratory failure. The baby was intubated, and mechanical ventilation was initiated to manage respiratory failure. Unfortunately, a sudden cardiopulmonary arrest occurred on the sixth day of life, as a result of respiratory failure due to severe airway obstruction, PDA, severe PPHN and sepsis. The infant died despite advanced cardiopulmonary resuscitation.

**Figure 3:**
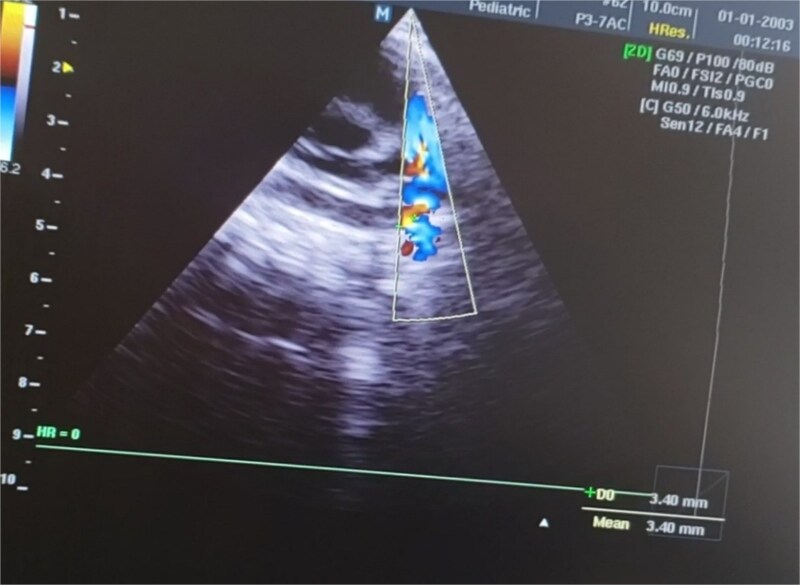
The Doppler echocardiography of the newborn baby shows blood flow from the aorta to the pulmonary artery, indicating a 3.4 mm patent ductus arteriosus. This anomaly contributed to the development of PPHN and hemodynamic instability of the infant.

**Figure 4:**
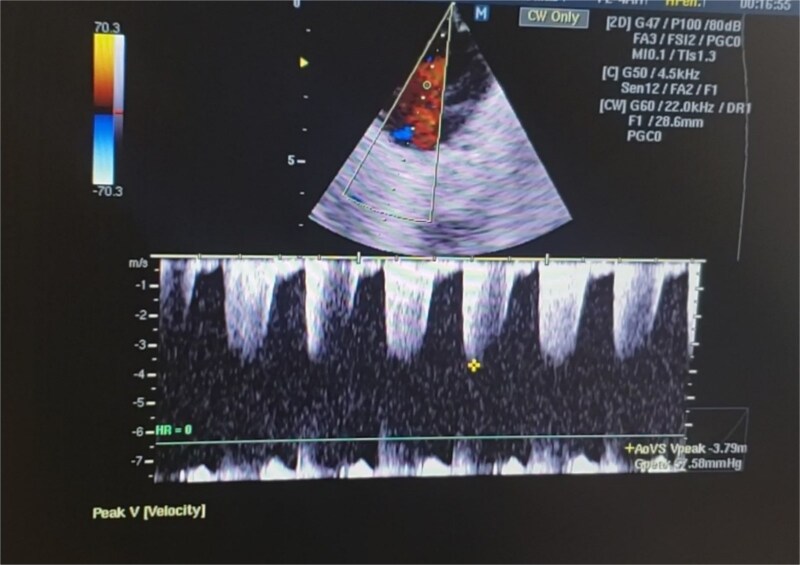
The echocardiography of the infant shows a severe PPHN with a peak pressure gradient of 67 mmHg. The combination of this disorder is associated with a worse prognosis of PRS.

**Figure 5:**
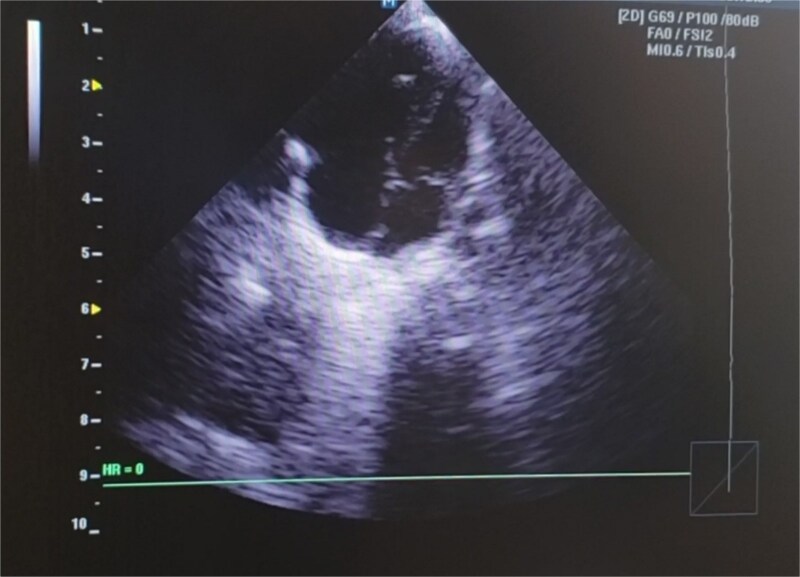
Dilated right atrium and ventricle are visible in the echocardiographic image of the index baby, which is mainly due to severe PPHN.

**Figure 6:**
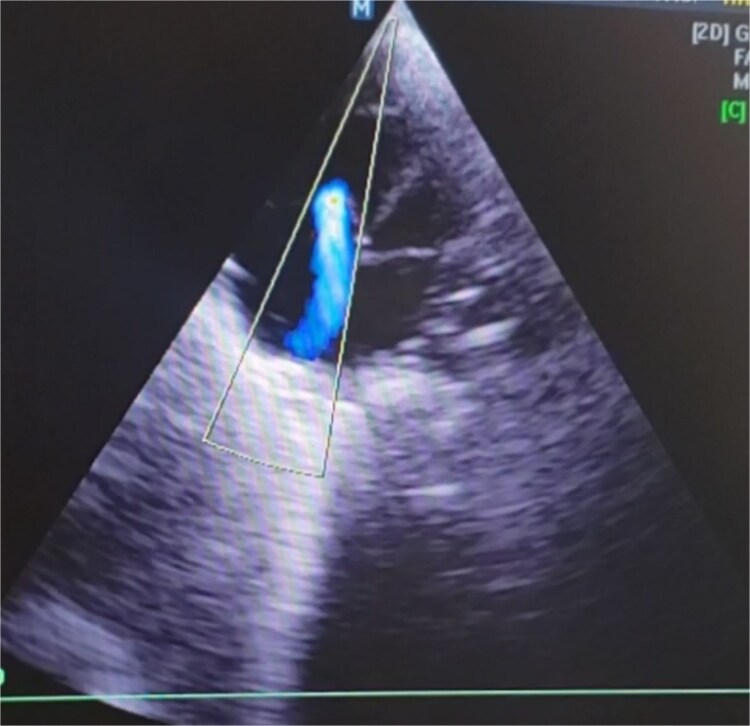
The echocardiographic image demonstrates severe tricuspid regurgitation. This pathological finding is usually associated with severe PPHN.

## Discussion

Pierre Robin, a French stomatologist, first reported the combination of micrognathia, glossoptosis, and airway obstruction in 1923. Later, this triad was called Pierre Robin syndrome. In the 1970s, the term ‘Pierre Robin syndrome’ was changed to ‘Pierre Robin sequence’ because the second term implies a set of morphologic defects that originate from a single, localized structural anomaly and lead to a series of subsequent defects [1]. The cause of PRS is unknown, and it can be isolated or part of a genetic syndrome [3, 5]. Micrognathia is an underdeveloped mandible characterized by a shorter body length and a greater angle. Glossoptosis is defined as moving the base of the tongue toward the pharynx. There is a broad spectrum of severity and associated respiratory distress [1, 5]. The clinical level of airway obstruction varies widely, from mild malfunction not requiring any intervention to severe respiratory distress necessitating intubation shortly after birth. Surgery is generally unnecessary for mild cases when conservative treatments are effective. Approximately 70% of PRS cases can be resolved by utilizing a prone and lateral positioning technique, which allows gravity to draw the tongue anteriorly and improve airway congestion. Another temporary method for maintaining an open airway is nasopharyngeal, oropharyngeal and laryngeal airways. Endotracheal intubation of the patient should be performed in refractory airway obstruction [3]. Surgery is needed to treat PRS cases that are more severe. Approximately ten percent of isolated PRS cases need to be surgically managed. Mandibular distraction osteogenesis to elongate the length of the mandible and alleviate tongue base obstruction, tracheostomy, and tongue-lip adhesion are surgical alternatives [6–8].

In newborn babies, an open ductus arteriosus following third day life is called a patent ductus arteriosus (PDA). The diagnosis is established by echocardiography [9, 10]. Symptomatic PDA has an adverse impact on respiratory outcomes, and its closure improves lung function. In term neonate, diuretic therapy and maintenance fluid restriction are used in the medical management of PDA [10, 11].

Persistent pulmonary hypertension of the newborn (PPHN) is characterized by a newborn’s persistently high pulmonary vascular resistance. Blood shunts through the ductus arteriosus and foramen oval from the right to the left as a result of pulmonary hypertension [12, 13]. PPHN typically appears soon after the birth, leading to severe hypoxia and respiratory distress. One of the most effective diagnostic methods is echocardiography [14]. PPHN is treated with respiratory support and pulmonary vasodilators to reduce pulmonary vascular resistance and decrease the severity of the right-to-left shunt. Inhaled nitric oxide and sildenafil are well-known pulmonary vasodilators [12, 13]. Neonatal sepsis is defined as a systemic inflammatory response syndrome caused by a proven or suspected infection. Fever, hypothermia, fast breath, tachycardia, chest retraction, leukopenia or leukocytosis, cyanosis, hypoxia, and an elevated C-reactive protein are all crucial diagnostic indicators. Supportive treatment and antibiotic therapy are the essential components of management [15, 16]. Newborns with a birth weight between 1500 and 2500 g are classified as low-birth-weight (LBW) infants, and those with an Apgar score below 7 at the fifth minute of life are considered to have perinatal asphyxia [15].

Based on the clinical manifestations, the current neonate was diagnosed as having PRS. Clinical diagnostic findings were micrognathia, glossoptosis and airway obstruction (Figs. 1 and 2). Stickler syndrome, Treacher Collins syndrome (TCS), ventricular septal defect (VSD), short stature, and cleft palate are common syndromes and anomalies associated with PRS [17]. Stickler syndrome was ruled out because the infant did not exhibit the clinical diagnostic signs of this syndrome, including flat midface, epicanthal fold, cataracts, joint hypermobility, and hearing loss [5]. Furthermore, the infant showed no diagnostic signs of Treacher Collins syndrome (TCS), including microtia, downward-slanting palpebral fissures, midface hypoplasia, cleft palate, or choanal atresia [18]. The doppler echocardiography of the infant detected a 3.4 mm PDA, severe PPHN with a pressure of 67 mmHg, dilated left ventricle and atrium, and severe tricuspid regurgitation (Figs. 3–6). Abnormal clinical and laboratory results, such as hypothermia, tachypnea, leukocytosis, an elevated level of C-reactive protein, an Apgar score of 6 at the fifth minute of life, and a birth weight of 2.3 kg, were used to diagnose neonatal sepsis, perinatal asphyxia, and LBW. The present case highlights the possible role of genetics in the etiology of PRS, as evidenced by the coexistence of CHD, the occurrence of PRS in the baby’s sister and parental consanguinity. In our hospital, genetic evaluation is not feasible. According to a systematic review, genetic mutations were noted in 30.9% of the 300 cases of PRS [19]. Although a sequence of developmental events is considered to be the cause of PRS, there is some evidence supporting a clear causal relationship between genetic variations and PRS [1, 5]. In 2023, multiple congenital and chromosomal anomalies with PRS were found in a study by Stoll et al., indicating the significant role of genetics in the pathogenesis of PRS [17]. These investigations validated our hypothesis regarding the important role of genetics in the development of PRS. The presence of PDA, PPHN, perinatal asphyxia, LBW, and sepsis alongside PRS in this case suggests that PRS may play a contributing role in the pathogenesis of these associated conditions. Infants with PRS may experience respiratory distress, cyanosis, apnea, and recurrent oxygen desaturations due to an airway obstruction close to the base of the tongue [18]. Airway blockage and hypoxia may cause pulmonary vasoconstriction and pulmonary hypertension [12, 14]. Amin et al presented a case of PRS with pneumonia and respiratory failure [20]. Furthermore, Biskup and Francis reported a case of PRS along with heart failure in an infant [21]. The studies of Gangopadhyay et al., Stoll et al., Soni et al., Amin et al., and Biskup and Francis supported our hypothesis that PRS may play a role in the development of PDA, PPHN, perinatal asphyxia and infection in neonates [5, 14, 17, 20, 21]. Thus, a thorough analysis is necessary to assess the relationship between PRS and PDA, PPHN, perinatal asphyxia, and infection. Based on the study of Logjes et al., about 10% of PRS are died in infancy, mainly due to respiratory insufficiency resulting from upper airway obstruction and associated condition [22]. The mortality rate among infants with PPHN varies globally. In developed countries, where advanced treatments such as iNO and ECMO are available, mortality rates are relatively low, between 7% and 9%. In contrast, studies from Asian countries report higher mortality rates, ranging from 12% to 46.4% [23]. However, severe PPHN was treated with sildenafil and respiratory support in this case. Unfortunately, our country lacks access to ECMO and iNO, the most efficient pulmonary vasodilator. The infant in this case experienced a sudden cardiopulmonary arrest and passed away on the sixth day of life due to respiratory failure caused by serious airway obstruction, PDA, severe PPHN, and sepsis. Consequently, it is crucial to detect coexisting conditions that may increase the infant mortality due to PRS, especially in settings with limited resources. The concurrent integration of PRS with PDA, severe PPHN, perinatal asphyxia, sepsis, and LBW in our case denotes a significant difference from the previously reported cases [3–5, 7, 14]. In the mentioned published case reports, PRS is accompanied by polysyndactyly, ectopic kidney, short stature, cleft palate, VSD, pneumonia, respiratory failure, heart failure and congenital anomalies of the eye, ear, face, and urinary and musculoskeletal systems. Therefore, the simultaneous combination of PRS with PDA, severe PPHN, perinatal asphyxia, sepsis, and LBW in our case highlights its uniqueness. Since such an integration has not been previously reported [3–5, 7, 14], this exceptional combination will provide new insights to the existing literature.

## Conclusion

The Pierre Robin sequence may increase the risk of infection, prenatal asphyxia, and PPHN. During the early neonatal period, its complications may compromise the lives of infants. Consequently, early detection and treatment of associated morbidities with PRS is crucial for reducing infant mortality, particularly in resource-constrained settings.
